# KRAS mutation analysis in ductal carcinoma of the pancreas; prognostic implications in elderly patients

**DOI:** 10.1186/1471-2482-13-S1-A31

**Published:** 2013-09-16

**Authors:** Alberto Oldani, Clemente De Rosa, Manuela Monni, Alfonso Terrone, Umberto Miglio, Marcello Garavoglia, Renzo Boldorini

**Affiliations:** 1 Department of Surgery University of Eastern Piedmont “Amedeo Avogadro, Hospital “Maggiore della Carità” Novara, Italy; 2 Department of Pathology University of Eastern Piedmont “Amedeo Avogadro, Hospital “Maggiore della Carità” Novara, Italy

## Introduction

Ductal adenocarcinoma of the pancreas is one of the highly aggressive tumors; the overall survival rate of less than 6% is probably due to biological rapid development and late tumor stage at the time of diagnosis [1].

Recently a progression model suggested that infiltrating pancreatic cancer arises from pancreatic duct lesions that shows to harbour activating KRAS point mutation [2].

Since 1988 KRAS is the most significant genetic alteration in pancreatic cancer and is considered as an early event in tumor progression [3].

In the present study we investigated a selected population of patients who underwent pancreaticoduodenectomy for pancreatic adenocarcinoma, with disease free resection margins, in order to evaluate whether a specific mutation could be related to a worst median survival time or onset of tumor recurrence and metastatisation.

## Methods

We analyzed a series of 27 patients aged more than 65 years (mean age 72, range 65 – 81), that underwent radical pancreaticoduodenectomy at our Institution for malignant pancreatic disease; all operations (pancreaticoduodenctomy following Whipple Child procedure and extended adenectomy) have performed by the same surgeon.

The inclusion criteria were:

- radical surgical procedure with complete tumor ablation

- resection margins without an histologically proven tumor involvement

- adequate period of follow up (at least 12 months)

Staging has been classified following TNM classification; grading has been assessed as well (G1), moderate (G2) and poor (G3) differentiated.

Haematoxilin –Eosin slides have been revised by the same pathologist in order to select the area with a percentage of tumor cells more than 70% and area of resection margin with normal cells.

DNA was extracted using EDTA – SDS/proteinase K followed by phenol – chloroform and resuspended with 30 µl of DEPC treated and RNAse free water.

KRAS was analysed by means of mutant enriched PCR (ME – PCR) to detect two hotspot in codon 12 and 13 of exon 2, that include more than 95% of mutations.

Fisher’s exact test was performed in order to evaluate correlations between survival and clinical and pathological findings; overall survival (OS) and disease free survival (DSF) curves have been carried out using Kaplan – Meier methods and compared with Log – Rank test.

Non – parametric t – student test and Mann Whitney test have been used in order to compare average and median OS between the groups analyzed.

Significant values were considered when p<0.05, with an interval of confidence of 95%.

## Results

No perioperative mortality occurred; postoperative morbidity rate was 7.41%(1 case of peritoneal bleeding needing re – operaton and 1 case of pancreatic fistula treated conservatively).

20 of 27 patients (74.07%) died with a mean time of 23 months after surgery (range 12 – 51 months).

12 patients (44.44%) died for liver metastasis, in 4 cases (14.81%) local recurrence occurred; 2 patients (7.41%) develop both local recurrence and liver metastases; brain metastases was observed in 1 case (3.70%) and peritoneal carcinomatosis in 1 case (3.70%).

No statistical correlation was found comparing survival data with gender, staging, grading and perineural invasion.

KRAS gene was successfully amplified with enriched PCR in all samples.

4 tumor samples (14.81%) showed a KRAS wild type, whereas the remaining 23 (85.19%) harboured mutations; in details: G12D (13 cases, 48.15%), G12R (4 cases, 14.81%), G12V (3 cases 11.11%), G12 A (1 case, 3.70%), G12C (1 case, 3.70%), G12S (1 case, 3.70%).

No mutations have been detected in codon 13.

Among the mutated patients, 4 had local recurrence (17.39%) and no statistical correlation was found between patients presenting the most common mutation G12D and patients with other mutations (P=.60).

OS of G12D patients was compared with OS of patients with other mutations by Kaplan – Meier curves without finding statistical significance.

Similarly, we performed Kaplan – Meier curves for testing whether disease free survival (DFS) could be statistically different between the G12 mutated group and the other patients: no significant results were obtained.

Since all the wild type patients (WT) died during the follow up, we compared OS of WT with those mutated in order to evaluate if mutated patients showed a better outcome, without finding any statistical correlation.

Despite the average OS and DFS seems to indicate a tendency of better survival for patients that did not harbour G12D mutation (31.5 ± 5.6 vs 25 ± 4.5 months), survival curves, t – student test and Mann – Whitney test did not show statistical significance (P=0.27, P=.38 and P=.3 respectively).

Finally, the comparisons of means and medians of the analyzed groups (G12D vs all patients; G12D vs other mutations patients and G12D vs WT patients) did not show statistical significance.

## Conclusions

Radical resection with adequate adenectomy is the gold standard surgical procedure and provides the only chance to increase survival in patients with pancreatic cancer; anyway, despite the improvement of surgical techniques and the use of adjuvant treatments, pancreatic carcinoma is still one of the five leading causes of cancer death worldwide, especially in elderly patients [4].

Molecular investigations of pancreatic tumor determined a progression model in which KRAS gene mutation is an early event in tumorigenesis, occurring in pancreatic duct lesions with minimal cytological and architectural atypia (PanIN 1A), and could be associated to prognostic implications, in order to provide a useful biomarker for pancreatic cancer [5].

Our experience demonstrated that, as reported in Literature, KRAS altered expression is very common in pancreatic cancer, occurring in more than 85% of cases; despite the small number of patients considered in our study (due to a selection conducted following strict criteria), we can conclude that no correlation between any subtype of KRAS mutation, OS and DFS exist; further investigations by enlarging patients series is needed.
